# Matrix Metalloproteinases in Inflammatory Bowel Disease: An Update

**DOI:** 10.1155/2015/964131

**Published:** 2015-04-08

**Authors:** Shane O'Sullivan, John F. Gilmer, Carlos Medina

**Affiliations:** School of Pharmacy and Pharmaceutical Sciences, Trinity College, Dublin, Ireland

## Abstract

Matrix metalloproteinases (MMPs) are known to be upregulated in inflammatory bowel disease (IBD) and other inflammatory conditions, but while their involvement is clear, their role in many settings has yet to be determined. Studies of the involvement of MMPs in IBD since 2006 have revealed an array of immune and stromal cells which release the proteases in response to inflammatory cytokines and growth factors. Through digestion of the extracellular matrix and cleavage of bioactive proteins, a huge diversity of roles have been revealed for the MMPs in IBD, where they have been shown to regulate epithelial barrier function, immune response, angiogenesis, fibrosis, and wound healing. For this reason, MMPs have been recognised as potential biomarkers for disease activity in IBD and inhibition remains a huge area of interest. This review describes new roles of MMPs in the pathophysiology of IBD and suggests future directions for the development of treatment strategies in this condition.

## 1. Introduction

Inflammatory bowel disease (IBD) which includes both ulcerative colitis (UC) and Crohn's disease (CD) is a chronic and relapsing autoimmune disease characterised by inflammation of the gastrointestinal tract. The estimated mean prevalence of IBD in western countries is 1 in 1,000 [1, 2] and although data are less available for the developing world, incidence of the disease is rising globally [3, 4]. Both idiopathic forms of the disease share common symptoms of abdominal pain, diarrhoea, rectal bleeding, and fever. Ulcerative colitis is characterised by continuous inflammation involving the rectum and colon which extends proximally. Crypt abscesses from infiltration of neutrophils and ulceration of the mucosa is observed. Crohn's disease may affect any region of the gastrointestinal tract intermittently with the terminal ileum being the most common. The inflammatory process may extend through the intestinal wall narrowing the intestinal lumen and is histologically characterized by the formation of granulomas, fibrosis, and fistulae [5, 6].

The human matrix metalloproteinases (MMPs) are a family of 24 zinc dependent endopeptidases. They are grouped by domain structure and substrate preference into collagenases, gelatinases, stromelysins, and membrane type MMPs (MT-MMPs) [7]. The subgroups of MMPs have distinct structural domains but all possess a conserved catalytic domain with a Zn^2+^ at the active site and a prodomain which confers latency. The family of proteases were first studied for their ability to degrade the extracellular matrix and basement membrane to facilitate cell migration, infiltration, and tissue remodelling. As our understanding of MMPs has grown, they have been recognised as key regulators of cell function through their ability to cleave a vast range of cytokines, chemokines, receptors, proteases, and adhesion molecules to alter their function [8, 9]. MMPs are regulated at several levels from transcription, translation, secretion, and activation. There is also a large list of physiological inhibitors of MMPs which serve to regulate MMP activity and proteolysis. The four tissue inhibitors of MMPs (TIMPs) are specific inhibitors of MMPs that reversibly inhibit the MMPs in a 1 : 1 stoichiometric fashion.

These enzymes have long been linked with IBD and their role in intestinal inflammation was reviewed by Medina and Radomski in 2006 [5]. Our current understanding of the aetiology of IBD is that genetic susceptibilities in gut barrier integrity and innate and adaptive immune response can lead to an inappropriate inflammatory reaction in response to bacteria in the gut or other environmental factors [10, 11]. It is in this context that we review the recent evidence for the role of MMPs in the disease.

## 2. Association between MMPs and IBD: Enzymes Involved and Cellular Source

Most MMPs are transcriptionally upregulated in response to proinflammatory cytokines, cell-cell, or cell-ECM interactions [12]. The collagenases (MMP-1, -8, and -13), gelatinases (MMP-2 and -9), stromelysins (MMP-3 and -10), matrilysin (MMP-7), and macrophage elastase (MMP-12) are the most studied in the context of IBD. The wide range of cellular sources, which has been known to include epithelial cells, mesenchymal cells, and leukocytes, has been reinforced in recent studies. Myofibroblasts are now recognised as playing an active role in intestinal inflammation and the pathogenesis of IBD. These stromal cells have been shown to secrete MMP-2 and, upon stimulation, MMP-1, -3 and -9 [13–15]. Human colonic epithelium was shown to produce increased amounts of MMP-1, -3, -7, -9, -10, and -12 in IBD patients [16] and mucosal biopsies from UC patients identified vascular endothelial cells and infiltrating leukocytes as the major sources of MMP-7 and -13 [17]. Infiltrating macrophages were seen to be a major source of MMP-8, -9, and -10 in human IBD and a mouse model of colitis [18, 19] and isolated IgG plasma cells from IBD patients were shown to produce high and sustained amounts of MMP-3 [20]. Neutrophils are also major contributors of MMP-9 in intestinal inflammation where it is stored in granules and can be released upon stimulation [18, 21].

The range of cell types that secrete MMPs during intestinal inflammation reflects their integral involvement in the pathogenesis of IBD. Several studies from the previous decade have suggested a role for MMPs in IBD by showing their transcriptional upregulation and increased activity during active inflammation in the gut. The evidence for the involvement of MMPs in human IBD is unequivocal and recent reports further describe instances, pattern of expression, and cellular sources of the MMPs.

Transcripts or protein levels of MMP-1, -2, -3, -7, -9, -10, -12, and -13 are demonstrated to be upregulated in inflamed IBD mucosa or serum of IBD patients and MMP proteolytic activity was increased in cells from inflamed IBD epithelium [16, 22–25]. Gene expression profiling showed that MMP-1 was upregulated in UC and CD and linked to HIF-1 mediated inflammation. MMP-3 and -7 were upregulated in UC and MMP-7 was associated with genes known to regulate angiogenesis [26, 27]. Other studies have focused on the involvement of groups or individual MMPs in IBD or models of colitis.

### 2.1. Gelatinases (MMP-2, -9)

MMP-9 mucosal expression and protein levels, as well as serum antigen levels were significantly higher in UC patients compared to controls and these levels corresponded to the severity of the disease. Interestingly, these trends were not replicated in lymphocytic colitis or collagenous colitis where MMP-9 does not seem to contribute to the severity of the disease [28]. Gene expression profiling combined with qPCR has shown the upregulation of MMP-2 in paediatric CD [29]. This enzyme was also shown to be upregulated in a rat TNBS-induced colitis model and corresponded with the severity of the disease [30] which is in agreement with earlier studies [31, 32]. A murine DSS induced colitis model showed increased gelatinase mRNA levels in the colon [33]. Furthermore, patients with ischaemic colitis show increased gelatinase expression in inflamed areas compared with noninflamed areas or control patients implicating them in inflammation [34] and gelatinase-double knockout mice were protected from DSS, TNBS, or* Salmonella typhimurium* induced colitis [35].

### 2.2. Stromelysins (MMP-3, -10)

The expression of MMP-3 has been shown to be significantly upregulated in inflamed areas of colons of IBD patients compared to uninflamed areas implicating its involvement in the inflammatory process [36]. In addition, increased expression of epithelial MMP-10 and stromal TIMP-3 has been found in both UC and CD paediatric patients compared to non-IBD patients [37]. The stromelysins were deemed to be the greatest contributors to DSS induced colitis in one study and their inhibition with siRNA or blocking of the signalling pathways leading to their upregulation resulted in an amelioration of colitis [38].

### 2.3. Collagenases (MMP-1, -8, -13)

MMP-1 has been shown to be upregulated in ulcerated and inflamed areas of colon mucosa of UC patients and its expression correlates with severity of inflammation [39]. MMP-1 and TIMP-1 plasma and colonic mRNA levels are increased in UC correlate with disease severity [40]. Expression is also greater in the inflamed areas of colons in UC patients and MMP-1/TIMP-1 ratio is a measure of inflammation [41]. A group studying the Na^+^/H^+^ exchanger (NHE3) discovered that NHE3^−/−^ mice developed spontaneous colitis restricted to the mucosa of the distal colon with a concomitant 15-fold increase in MMP-8 expression [42]. MMP-13 has also shown to be present in the inflamed areas of colon in IBD patients but absent in noninflamed colons or in acute diverticulitis and MMP-13 expression correlated with histological measures of disease [43].

### 2.4. Macrophage Elastase (MMP-12)

MMP-12 was also shown to be upregulated in IBD patients as well as T-cell mediated model of colitis and contribute to epithelial degradation and MMP-12^−/−^ mice were protected against TNBS induced colitis [44]. Epithelial and stromal MMP-12 along with MMP-3 and -7 have been also upregulated in pouch mucosa of paediatric onset UC, suggesting that the expression of MMPs paediatric UC pouch in the long-term shares characteristics with IBD [45].

## 3. Genetic Basis of IBD: MMP Polymorphisms

Susceptibility to the development of IBD is associated with polymorphisms in genes coding for elements of the immune system or epithelial barrier. First degree relatives of IBD patients have an increased relative risk of up to tenfold compared with background population [46–48]. NOD2 (CARD15/IBD1) has been identified as a susceptibility gene for CD which can affect host interaction with LPS and trigger NF-*κ*B signalling [49–51]. Several large genome wide studies have identified loci that are linked to IBD; 16q12 (IBD1), 12q13 (IBD2), 6p21 (IBD3), 14q11 (IBD4), 19p13 (IBD5), 5q31-q33 (IBD6), and Xq21.3 [52–57] and polymorphisms of genes other than NOD2 have been identified as conferring susceptibility including IL23R and ATG16L1 [58–60]. Given the recognised involvement of MMPs in IBD and the strong genetic component of the disease, several groups have investigated the associations between known MMP polymorphisms and IBD phenotypes.

An extensive study of MMP-1, -2, -3, -7, -8, -9, -10, -12, -13, and -14 and TIMP-1, -3, and -4 single nucleotide polymorphisms (SNPs) in UC, carried out in a New Zealand cohort, found that SNPs in MMP-3, MMP-8, MMP-10, and MMP-14 were associated with the disease [61]. The study was able to make associations with some of the SNPs and disease phenotype but the associations made with UC were not replicated in a Dutch cohort. Primary sclerosing cholangitis (PSC) is a cholestatic liver disease characterised by chronic inflammation and fibrosis. It is believed to share pathologies with IBD and 50–80% of PSC patients also suffer from IBD [62]. Polymorphisms of the MMP-3 gene have been associated with PSC and with UC where the mechanisms are likely to be the same but have yet to be determined [63–65]. An MMP-3 SNP has also been associated with increased risk of stenosing behaviour in CD [66]. Preliminary studies have shown associations between collagenous colitis and an MMP-9 SNP but not MMP-1 or MMP-7 SNPs [67]. Two different TIMP-1 SNPs were associated with increased susceptibility to CD [66].

## 4. Role of Bacteria in IBD: Interactions with MMPs

There is a body of evidence to suggest that gut microbes are the key to the initiation and development of IBD. It is likely that the disease is triggered by interaction of the gut microflora with host defences following impaired barrier function. Antibiotics or some probiotics have shown to be of benefit in treating IBD [68, 69]. Further evidence for the role of bacteria in triggering the disease is that gnotobiotic mice do not develop colitis but it rapidly emerges when normal luminal flora are reintroduced [70, 71] and that experimental colitis can be induced in mice in response to adherent-invasive* E. coli*, strains of* Salmonella* [72] or* Helicobacter* [73, 74].

Much interest has been generated over the years in specific species of bacteria which may be causative agents for IBD. Mycobacterium avium paratuberculosis (MAP) has sparked much recent debate regarding its involvement in Crohn's disease and is the subject of numerous reviews and meta-analyses [75–84]. The subspecies is the causative agent of Johne's disease, an inflammatory disease mainly in ruminants with similarities to CD such as diarrhoea, leukocyte infiltration to the intestinal wall, and intestinal lesions. Indeed, MAP has been shown to be present in a higher percentage of IBD patients than healthy controls; however, the associations have not proven to be conclusive [77, 80]. MAP infection is associated with an upregulation of MMPs in cattle and it upregulates MMPs in cultured murine macrophages [85–87]. A recent study examined the expression of MMPs in UC patients who tested positive for MAP DNA but found it no different to patients without MAP DNA [88]. In contrast to this, another group showed that mice given oral MAP had increased colonic expression of MMP-2, -9, -13, and -14 as well as TIMP-1 in response to the bacteria [89].

A combination of the antibiotic minocycline and the probiotic* E. coli Nissle 1917* was shown to improve recovery form DSS induced colitis in mice including improved ratio of beneficial/harmful bacteria and reduced MMP-9 expression [90]. However, no experiments were carried out to discern the antibiotic effects of minocycline from its MMP inhibitory and immunomodulatory effects in reference to the protective effect observed. A group studying* Citrobacter rodentium*-induced colitis in mice found that MMP-9 was upregulated in the model. While epithelial barrier integrity and histopathological observations were unchanged between MMP-9^−/−^ and wild type mice, increased IL-17 expression was observed in the MMP-9^−/−^ mice. Interestingly, the gut microbiome was altered in wild type mice following infection but not in MMP-9^−/−^ mice, implicating a role for MMP-9 in the depletion of microbial diversity in the gut, after infection [91]. MMP-7 can also modulate the gut microbiome where it has been shown to cleave the inactive alpha-defensin, procryptdins, to their active form [92]. Cryptdin-4 is mostly active against noncommensal bacteria; however, its reduced form, which is inactivated by MMP-7, demonstrates greater bacteriocidal activity against commensal gut bacteria [93].

Interestingly, direct antibacterial effects for MMP-12 have been demonstrated as it can disrupt bacterial cell membranes in the macrophage phagosome [94].

## 5. Recent Pathways Regulating MMPs in IBD

**Figure 1:**
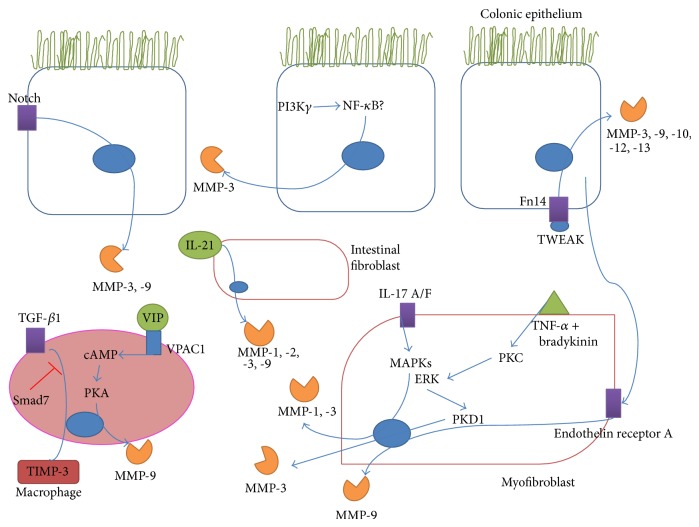
Recently described signalling pathways in the gut leading to the upregulation of MMPs in IBD or models of colitis. The various mediators whose interaction with receptors on colonic epithelial cells, intestinal fibroblasts, myofibroblasts, and macrophages can trigger signal transduction pathways leading to increased expression of MMPs or TIMPs are shown. PI3K*γ* (phosphatidylinositol-3 kinase *γ*), NF-*κ*B (nuclear factor *κ*B), TWEAK (TNF-related weak inducer of apoptosis), TGF-*β* (tissue growth factor *β*), cAMP (cyclic adenosine monophosphate), PKA (protein kinase A), VIP (vasoactive intestinal peptide), VPAC1 (vasoactive intestinal peptide receptor 1), MAPKs (mitogen activated protein kinases), ERK (extracellular signal-regulated kinase), PKC (protein kinase C), PKD (protein kinase D), and IL (interleukin).

MMPs are regulated at several levels from transcription to enzyme activation. The interconnectedness of inflammatory networks including activation of signal transduction pathways, where release of a cytokine can trigger an inflammatory cascade, or indeed the protease web where activation of a proenzyme can in turn lead to the activation of a host of other enzymes and their targets, makes delineation difficult. Despite decades of study, the place and role of MMPs in this network is still under investigation and here we summarize the recent studies of the place of MMPs in this network in IBD (Figure 1).

Some of these studies have shown new inducers of MMPs in IBD. IL-17A and IL-17F can increase secretion of MMP-1 and -3 in subepithelial myofibroblasts and also enhance the actions of IL-1*β* and TNF-*α* on these MMPs in a MAPK mediated manner [95]. IL-21 was also shown to play a part in the upregulation of MMP-1, -2, -3, and -9 in intestinal fibroblasts without an increase in TIMPs [96]. TNF-like weak inducer of apoptosis (TWEAK) is a member of the TNF-family of cytokines that signals through its receptor, fibroblast growth factor-inducible molecule 14 (Fn14). Inhibition of the TWEAK pathway resulted in reduced severity of TNBS induced colitis in mice resulting in reduced expression of MMP-3, -9, -10, -12, and -13 along with other inflammatory mediators [97].

Other recent studies have characterised various new signalling pathways involved in the transcriptional upregulation of MMPs in intestinal inflammation. For example, inhibition of Notch signalling reduces MMP-3 and -9 expression in DSS induced colitis [98]. A recent study showed that inhibition of PI3K*γ* had anti-inflammatory effects in TNBS induced colitis which resulted in increased Treg response and decreased NF-*κ*B mediated expression of MMP-9 and other inflammatory mediators [99]. VPAC1, a receptor for vasoactive intestinal peptide (VIP), enhances DSS induced colitis through activation of PKA and increased MMP-9 expression, among other mediators [100]. Colonic myofibroblasts were shown to produce MMP-3 in response to bradykinin and TNF-*α* through a pathway that involved activation of PKC and ERK, establishing a critical role for the downstream PKD1 [15]. A study investigating the crosstalk between subepithelial myofibroblasts and colonic epithelial cells found that the myofibroblasts produced MMP-9 in response to the cytokines TNF-*α*, IL-1*β*, and TGF-*β* but was inhibited by IFN-*γ*. Interestingly, incubation of the myofibroblasts with media containing the releasate of cytokine stimulated epithelial cells resulted in an upregulation of MMP-9 which was mediated by endothelin receptor A signalling [14]. TGF-*β*1 was shown to protect against TNBS induced colitis in mice through upregulation of TIMP-3 in lamina propria mononuclear cells. Knock-down of Smad7, the TGF-*β*1 receptor antagonist, resulted in increased TIMP-3 expression [101]. Chymase has been shown to be a relevant activator of pro-MMP-9 in DSS induced colitis [102]. In a study on the effects of mast cell tryptase in IBD, MMP-3, -9, and -13 were downregulated in DSS treated mast cell protease (MCP) 6/7^−/−^ mice compared to wild type DSS treated showing a regulation of these MMPs by the proteases [103]. All these studies contribute to our understanding of the signalling pathways involved in MMP regulation in IBD.

## 6. New Evidence for Functions of MMPs in IBD

**Figure 2:**
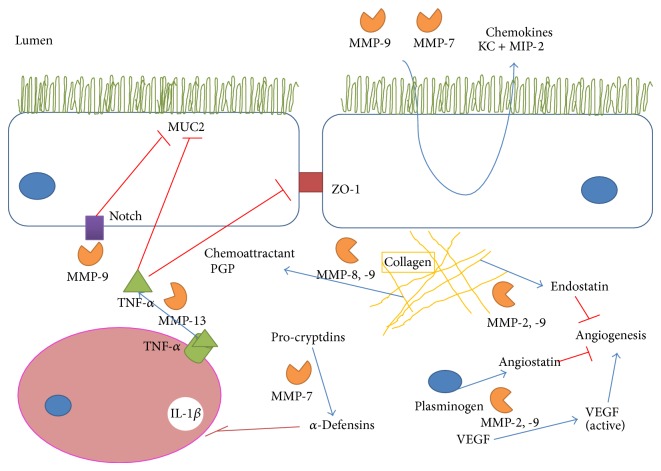
Recently described functions for MMPs in IBD. A summary of some of the roles of MMPs in IBD is shown including regulation of epithelial barrier through MUC2 expression and activation of immune response through cleavage of procryptdins, chemokines, and cytokines. MMPs also have a role to play in angiogenesis through allowing migration of endothelial cells and alteration of VEGF but also release of endostatin and angiostatin. MUC2 (mucin 2), KC (CXCL1), MIP-2 (macrophage inflammatory protein 2), ZO-1 (zona occludens protein 1), VEGF (vascular endothelial growth factor), and PGP (proline-glycine-proline).

As stated previously, the view of MMPs as simple ECM proteases is vastly oversimplified. MMPs can activate or inhibit a wide range of cytokines, chemokines, receptors, adhesion molecules and signalling molecules in order to regulate local inflammation in the gut and new roles are being discovered continually (Figure 2). Here we review the recent literature concerning MMP action in intestinal inflammation beyond ECM degradation. A summary is provided in Table 1.

### 6.1. MMPs in the Immune Response

The *α*-defensins, which modulate IL-1*β*, are cleaved and activated by MMP-7 [104]. MMP-7^−/−^ mice were more susceptible to DSS induced colitis. MMP-7 is postulated to reduce IL-1*β* release through activation of *α*-defensins [105]. Another study highlights the dual effect of the protease in colitis where it mediates both tissue injury and also healing in DSS induced colitis. MMP-7^−/−^ mice had a lower mortality rate and the increased inflammation in the wild type animals was ascribed to increased neutrophil migration through increased expression of the chemokines KC and MIP-2 [106]. Transgenic mice overexpressing epithelial MMP-9 developed worse DSS and ST induced colitis which correlated with an increased expression of KC [107]. An interesting recent study described how MMP-10^−/−^ mice developed a more severe colitis in response to DSS and that MMP-10 derived from macrophages was required for gut healing. Although the mechanisms of this protection were not described, depleted macrophage numbers in the MMP-10^−/−^ mice may have prevented colonic healing [19]. Proline-glycine-proline (PGP) is a product of collagen breakdown by propyl endopeptidase (PE) and MMPs and is known to be a chemoattractant for neutrophils [108]. It was recently demonstrated that this is a novel mechanism for MMP induced neutrophil infiltration in IBD where MMP-8, -9, and PE were upregulated in the inflamed intestines of IBD patients and in mice with DSS induced colitis. Generation of these enzymes resulted in PGP and increased infiltration of neutrophils. PGP neutralisation resulted in decreased neutrophil infiltration and lessened the severity of the colitis [18].

TNF-*α* is a proinflammatory cytokine whose levels are increased in the blood, colonic mucosa, and stools of IBD patients. It contributes to the pathogenesis of the disease by increasing inflammation through MAPK and NF-*κ*B activation, increasing cell proliferation and altering epithelial barrier permeability [109]. Anti-TNF therapy has been a major breakthrough in recent years for the treatment of moderate to severe CD and UC refractory to traditional therapies. Upon synthesis, homotrimeric TNF-*α* migrates to the cell membrane where it is cleaved into the soluble and biologically active form. Until recently, TACE/ADAM17 was believed to be the only relevant in vivo activator of TNF-*α* [110]; however, MMP-13 has now been demonstrated to perform the same function. Vandenbroucke et al. discovered that the observed effects of MMP-13 on epithelial integrity in DSS induced colitis, such as mucus depletion, intestinal inflammation, and loss of tight junction function were mediated through activation of TNF-*α* [111]. The significance of this discovery in development of a therapy will remain to be seen.

### 6.2. MMPs in Angiogenesis

Angiogenesis is now believed to play a major role in the process of chronic inflammation and has been suggested to contribute to the pathology of IBD. Early cytokine, chemokine, and growth factor release facilitate the process which promotes increased leucocyte infiltration. The understanding of angiogenesis in IBD was summarised previously [112]; here we limit ourselves to the more recent studies where MMPs are implicated.

Endothelial cell-produced MMP-1, -3, and -9 are upregulated in human IBD and experimental colitis. These enzymes are potentially involved in different aspects of angiogenesis. MMPs could have dual roles in angiogenesis acting as proangiogenic mediators during tissue remodelling and then as antiangiogenic mediators through generation of angiostatin preventing vessel maturation. At a simplified level, MMPs facilitate angiogenesis through remodelling of the ECM, permitting incorporation of migrating endothelial cells which then form new vessels [113]. While this may be accurate, regulation of angiogenic factors in the gut by MMPs is also likely to contribute. It is known that MMPs can release bound forms of VEGF-A from the ECM, cell membrane, heparin affinity regulatory peptide (HARP), and connective tissue growth factor (CTGF), although the functional relevance of this has been assessed in cancer models and not in IBD [8]. MMP-1 and -3 can cleave heparin-sulfate proteoglycan in endothelial cells to release basic fibroblast growth factor (bFGF) [114] and transforming growth factor-*β* (TGF-*β*) can also be released and activated by MMPs [115–117]. Further evidence for the roles of MMPs in angiogenesis is reviewed by Rodríguez et al. [8].

Cleavage of collagen XVIII and plasminogen by several MMPs can generate endostatin and angiostatin, respectively, which are both antiangiogenic factors [118]. These antiangiogenic factor levels were upregulated in a rat model of UC. Administration of mesalamine was able to restore the proangiogenic balance by inhibiting gelatinases and thus generation of these fragments [119].

Others argue that increasing VEGF can lead to excessive and pathological angiogenesis in IBD [120–122] and that angiogenesis blockade may even be viable as a therapeutic strategy for reducing disease severity in IBD [123]. In this context, the increasing levels of MMP-9 lead to an increased endostatin concentration and treatment with endostatin in MMP-9^−/−^ mice can reduce the severity of colitis [124] indicating a protective role for the enzyme. The ability of MMP-9 to release bound VEGF-A and alter its angiogenic outcome [125, 126] was not assessed in the context of IBD in the studies described; however, serum MMP-9 levels were found to correlate with serum VEGF in CD but not UC patients [127]. The studies of the regulation of angiogenesis by MMPs in IBD are limited in scope but provide evidence that many of the discoveries made in cancer and other diseases would also hold true for IBD. The net contribution of the MMPs will depend on the microenvironment and thus the generation of pro- or antiangiogenic factors.

### 6.3. MMPs and Epithelial Barrier Function

Epithelial barrier integrity is essential in maintaining intestinal homeostasis. Infiltration of luminal contents into the lamina propria triggers a local inflammatory response leading to release of proinflammatory mediators, release of MMPs, and further epithelial degradation and inflammation. Indeed, leaking of bacteria or alarmins into the bloodstream can trigger a systemic response, sepsis, or multiorgan failure [128]. MMPs have been implicated in modulation of the epithelial barrier elsewhere in the body [129–131] and owing to its importance in intestinal inflammation, also in the gut, as discussed below.

When investigating the effect of MMP-9 on the colonic epithelial barrier in a model of colitis, it was found that MMP-9^−/−^ mice had increased goblet cell numbers and increased MUC2 expression. Overexpression of MMP-9 resulted in a decrease in goblet cell differentiation [107] and thus decreased MUC2 expression. This reduction of MUC2 expression reduces the protective mucin barrier and was shown to affect the adherence of* Salmonella typhimurium* [132]. The tight junction protein claudin-1, which has been previously implicated in colitis associated cancer (CAC) [133], was shown to be involved in epithelial homeostasis. Upregulation of claudin-1 following DSS induced colitis results in an upregulation of MMP-9 which triggered Notch signalling resulting in decreased MUC2 expression through decreased goblet cell differentiation [134]. It was also shown that the gelatinases play opposing roles in intestinal inflammation where MMP-9 can potentiate colitis but MMP-2 participates in maintaining epithelial barrier function to prevent the initiation of colitis [135]. MMP-13 indirectly regulates epithelial barrier function through activation of TNF-*α*. Activation of the cytokine increases intestinal epithelial permeability by mediating the endocytosis of the tight junction protein ZO-1 and reducing MUC2 expression, effects that are absent in MMP-13^−/−^ mice [111].

In a DSS induced model of chronic colitis described previously, wild type animals recovered more quickly and completely from the colitis than MMP-7^−/−^ animals which may be due to decreased neutrophil infiltration to the mucosa [106]. MMP-7 was shown to be hugely upregulated in an intestinal epithelial wound healing model and resulted in faster resolution of the wound. Under inflammatory conditions, simulated by addition of TNF-*α* and IL-1*β*, the expression levels were increased further which delayed wound healing [136]. This observation is likely applicable to many of the MMPs, where physiological roles contribute to the pathology of IBD under inflammatory conditions. Another study showed the ability of MMP-7 to cleave galectin-3 and to reverse its wound healing abilities [137]. The contribution of this effect in vivo is undetermined but adds a further layer of complexity.

### 6.4. MMPs in Intestinal Inflammation-Induced Fibrosis

Fibrosis is a pathological accumulation of ECM which occurs in the intestine as a consequence of IBD and has been recently reviewed [138]. MMP expression, and the balance between their levels and those of the TIMPs or other inhibitors, is crucial for normal ECM homeostasis. A disruption of this balance may promote fibrosis in the intestine. Despite therapeutic advances in IBD, none prevent or reverse established strictures. In UC, fibrosis will normally affect the mucosa and submucosa whereas in CD, transmural thickening can lead to stricture and require surgery [139]. In humans, TGF-*β*/Smad pathway seems to be a major contributor to fibrosis in the gut where it can inhibit MMPs and increase the production of TIMPs in mucosa overlaying strictures and in cultured myofibroblasts [96, 140] We have also found that the TGF-*β*/ALK5/Smad pathway participates in the pathogenesis of experimental intestinal fibrosis. Indeed, upregulation of ALK5 and TIMP-1, phosphorylation of Smad2 and Smad3 proteins, and increased intestinal wall collagen deposition were found in anaerobic bacteria- and TNBS-induced colitis [141]. In addition, the antifibrotic effects of glutamine in TNBS induced colitis was partly attributed to abrogation of the overexpression of TGF-*β*, phosphorylated Smad3, and TIMP-1 [142].

IL-13 is also said to play a role in fibrosis elsewhere in the body partly through regulation of MMP-1 and TIMP-1 expression [143, 144]. A recent study showed that the cytokine can inhibit expression of MMP-1, -2, and -9 in cultured fibroblasts and that MMP-2 synthesis is not coordinately upregulated along with that of collagen in fibrotic CD colons [145]. In a DSS model of chronic colitis, the increased expression of gelatinases was said to protect against fibrosis through collagen degradation which is another protective role for these enzymes [33].

### 6.5. MMPs in Colitis Associated Cancer

Chronic inflammation plays a critical role in gastrointestinal carcinogenesis. As examples, chronic hepatitis, Barrett's oesophagus and IBD. Indeed, patients suffering from IBD are at higher risk for developing colonic neoplasia than normal population, particularly those with extensive colorectal inflammation (pancolitis) which continues for longer periods of time [146]. Indeed, colorectal cancer accounts for one sixth of all UC-related deaths [147]. The involvement of MMPs in colorectal cancer and metastasis has been extensively studied [148]. We have also found that MMP-9 upregulation is an early event in the adenoma-carcinoma sequence and, therefore, MMP-9 might be a molecular marker for early colorectal carcinogenesis [149]. However, colitis-associated colorectal cancer (CAC) does not follow an adenoma-carcinoma sequence which is initially associated with genomic instability and the concomitant loss of key tumour suppressor genes. In contrast, CAC shows an inflammation—dysplasia—carcinoma sequence, in which a p53 mutation plays a key role in the early stage and later the APC function is diminished [150]. The p53 functions as tumour suppressor; therefore, the loss or mutation of p53 could lead to neoplasia formation.

**Figure 3:**
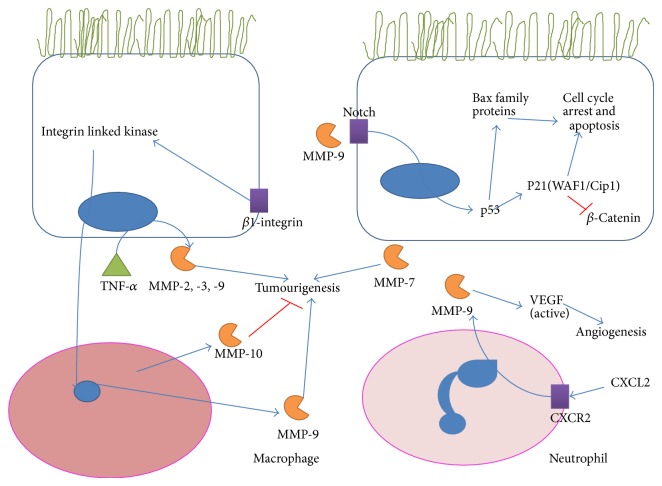
MMPs in colitis associated cancer. A summary of the reported involvement of MMPs in colitis associated cancer (CAC) including the dual roles of MMP-9 where it increases apoptosis and cell cycle arrest through notch cleavage and activation of p53 but also promotes tumour growth, partially through activation of vascular endothelial growth factor (VEGF). MMP-7 is upregulated in UC associated dysplasia and MMP-2 and -3 have also been reported to increase tumour growth. MMP-10 from macrophages is believed to inhibit colitis associated cancer based on a knock-out mouse study.

A summary of the role of MMPs in CAC is provided in Figure 3. It is interesting to note that while colitis is mediated by MMP-9, the same enzyme may play a protective role in CAC. In fact, MMP-9 could play dual roles in CAC. It has been shown that MMP-9^−/−^ mice are more susceptible to CAC than wild type mice and the protective effect of MMP-9 is believed to be mediated through Notch-1 activation and a subsequent decrease in *β*-catenin [151]. The same group further investigated these effects and concluded that the upregulation of MMP-9 in colitis leads to activation of Notch-1, increased p53 expression leading to increased levels of p21^Waf/Cip1^, and members of the Bax family proteins to resulting in cell cycle arrest and apoptosis [152].

In contrast to the protective effects of MMP-9, a mouse model of CAC found that activation of neutrophils by the chemokine CXCL2 induced MMP-9 expression which promoted neovascularization and possibly drove CAC [153]. Where integrin linked kinase (ILK) has been implicated in carcinogenesis, ILK-intestinal epithelial cell knockout mice showed reduced tumour growth and MMP-9 expression in experimentally induced CAC [154]. In addition, it has been also shown that infliximab, an anti-TNF-alpha antibody, could prevent CAC in DSS-induced colitis, an effect which was accompanied by the reduction of MMP-9 and MMP-11 levels [155]. Similar results were observed when omeprazole exerted a proapoptotic effect in a model of CAC and inhibited MMP-9 and -11 and MT1-MMP [156] and celecoxib reduced gelatinase colon levels in models of CAC [157].

Examination of human UC biopsy samples revealed that there was a direct correlation between the expression of MMP-7 and the grade of UC-associated dysplasia or carcinoma [158]. Further study is required to uncover the precise role of the protease in the progression of the disease. On the other hand, MMP-10 seems to play a protective role from CAC development as it has been found that MMP-10^−/−^ mice had significantly worse inflammatory scores and also higher propensity for development of dysplastic lesions after DSS exposure [19]. More research is required to discover the expression patterns of specific MMPs at the various stages of CAC, to reveal the roles that the enzymes play and the net contribution to tumorigenesis. This may then facilitate successful targeting of specific enzymes as a treatment.

## 7. MMP Inhibition

Failure of MMP inhibitors (MMPIs) in cancer trials led to a major rethink of the potential of these compounds in the clinic, but we can now reflect on how little was known about the functions of the specific MMPs in a given setting and the clinical effect of inhibition. Early phase I trials revealed unexplained musculoskeletal pain and inflammation which limited the dosage that could be administered. Phase II/III trials examining efficacy, were met with further problems. Owing to the fact that the MMPIs were cytostatic and not cytotoxic, conventional measures of efficacy such as reduction in tumour size were not appropriate. Chosen endpoint measures such as reduction in serum biomarkers were criticized for not necessarily reflecting any reduction in tumour growth and also for being unable to demonstrate MMP inhibition [159]. Ultimately, the trials failed to demonstrate efficacy and several were abandoned. Many researchers now agree that invasive or metastatic cancer may not have been appropriate diseases to trial of the drugs and that preclinical studies suggest the drugs may be more useful in treating earlier stage cancers or inflammatory conditions [160]. A review by Hu et al. covers the history and considers the future for MMP inhibition in the treatment of cancer and inflammation [12]. With continuous studies teasing the physiological from the pathological roles of MMPs in the setting of IBD, we are getting closer to being able to imagine clinical use of an MMPI in IBD. Here we summarise some of the reports of MMP inhibition in intestinal inflammation since 2006 (Table 2).

### 7.1. Synthetic MMP Inhibitors

Various studies have examined the potential of novel small molecule inhibitors of MMPs in animal models of colitis. Barbiturate-nitrate hybrids can inhibit MMP-9 activity partly through their nitric oxide mimetic properties [161]. NO mimetics may have the paradoxical effect of reducing NO/iNOS activity through negative feedback, therefore, reducing inflammation and associated MMP-9 release. Our group has recently reviewed the complex interactions of NO and MMP-9 and exploiting these interactions may be therapeutically beneficial in IBD [162]. Integration of a nitrate group as an NO donor may be a novel approach to enhancing efficacy and reducing side-effects of small molecule MMP inhibitors. The benefits of the MMPI RO28-2653 in the DSS model were attributed to its gelatinase selectivity. In particular, ability to spare MMP-1 and -7 reduced the side-effects observed with broad spectrum inhibition and efficacy was comparable to that of doxycycline [163]. Novel synthetic curcuminoid pyrazole derivatives inhibit MMP-9 activity in TNF-*α* and IL-1*β* stimulated Caco-2 cells [164]. Sequestration of MMP-2 by hydroxamate beads can inhibit MMP-2 activity and prevented disruption of the epithelial barrier in an in vitro model and is an interesting approach to MMP inhibition at the membrane [129]. The thalidomide analogue, CC-10004, was shown to reduce TNF-*α* and MMP-3 levels from mononuclear cells isolated from the lamina of CD patients [165].

Vitamin D has inherent immunomodulatory properties and synthetic analogues reduce hypercalcaemia. One such compound, ZK156979, has been shown to be effective in preventing TNBS induced colitis [166] and was shown to inhibit gelatinase activity in cultured peripheral mononuclear cells from healthy and IBD patients [167]. ZK191784 is an intestine specific vitamin D analogue which can exert an anti-inflammatory effect without causing hypercalcaemia [168–170]. This compound and calcitriol were tested in cultured colon biopsies of healthy and IBD patients and were found to reduce MMP-2, -3, and -9 levels as well as the adhesion molecules ICAM-1 and MAdCAM-1 [171].

Several studies have tested known MMPIs in models of human IBD or assessed the ability of therapeutics used in IBD to inhibit MMPs. The tetracycline antibiotics have long been known for their anti-inflammatory properties and for their ability to inhibit MMP expression. Minocycline was shown to reduced inflammation in DSS or TNBS models by reducing the expression of iNOS, proinflammatory cytokines, and MMP-2, -3, -9, and -13 [172] with greater benefit seen in DSS induced colitis when it is combined with the probiotic* E. coli Nissle 1917* [90]. The first generation hydroxamate MMPI ilomastat was shown to protect rats from TNBS induced colitis by inhibiting MMP-1 expression in the colon [173]. Slow release granules of mesalazine were able to reduce MMP-2 expression and inflammation [30]. A novel mechanism was described for 5-ASA in angiogenesis during UC where inhibition of TNF-*α* and gelatinase levels, reduced the levels of angiostatin and endostatin [119].

The effects of the clinically used infliximab on gelatinase levels in CD patients were examined and found that MMP-9 serum levels were consistently decreased following infliximab treatments and MMP-9 expressing polymorphonuclear leukocytes were also reduced in biopsy samples [174]. MMP-3 and -12 were decreased following 10 weeks of infliximab treatment in CD patients that were responders to the treatment [175]. Using mucosal explants of IBD and control patients, infliximab was shown to downregulate MMP-1, -3, and -9 levels as well as reducing their increase in response to pokeweed mitogen in a genotype dependent manner following analysis of TNF, MMP, and TIMP SNPs [176]. It has also been shown that infliximab could reduce the incidence of tumour development through reduction of MMP-9 and -11 in DSS-induced colitis [155]. Treatment of CD patients with anti-TNF therapy (infliximab or adalimumab) or with corticosteroids and other immunosuppressives (methotrexate or azathioprine) resulted in a decrease of epithelial MMP-7 and stromal MMP-9 and -26 and TIMP-1 and -3 [177]. In a similar study, the same group showed results in paediatric IBD where glucocorticoid therapy reduced serum MMP-7, TIMP-1, and MMP-7/TIMP-2 and anti-TNF therapy reduced MMP-7 but to a lesser extent. Interestingly, MMP-8 and -9 levels were not statistically significantly altered [178]. A larger study used microarrays to measure the mucosal expression of 24 MMPs along with TIMPs, ADAM(T)s, and growth factors. Most MMP expression was increased in IBD patients with the exception of MMP-28 which was downregulated and responders to infliximab had a gene expression and gelatinase activity was restored to control levels following treatment [179].

### 7.2. Natural Products

Research is increasing in the field of natural products as medicines and many of these have been directed towards MMP inhibition in intestinal inflammation.

The coumarin, auraptene, is found in several citrus fruits and has been shown to inhibit MMP-7 activity following DSS induced colitis [180]. The naturally occurring coumarin, 4-methylesculetin, showed comparable effects to sulphasalazine and prednisolone in TNBS induced colitis where it was able to inhibit MMP-9 [181]. 2′,4′,6′-Tris (methoxymethoxy) chalcone (TMMC) protected against TNBS induced colitis and was able to inhibit MMP-7 upregulation induced by TNF-*α* in HT-29 cells [182]. Curcumin is a component of turmeric with known anti-inflammatory properties and one group investigating its use in IBD showed that it could reduce MMP-3 among other mediators in ex vivo cultured colonic myofibroblasts from IBD patients [183]. PPGs and verbascoside were shown to reduce inflammation, pro-inflammatory signalling and gelatinase expression in DNBS induced colitis [184, 185]. Neovastat, a product found in shark cartilage, is a known inhibitor of MMPs and angiogenesis and has undergone clinical trials for the treatment of renal carcinoma and plaque psoriasis [186, 187]. More recently, this agent has shown to inhibit intestinal inflammation in TNBS induced colitis through inhibition of gelatinase expression [188].* Cordyceps militaris* is a traditional medicine widely used in East Asia to treat inflammatory conditions and has been shown to inhibit disease activity, along with iNOS and MMP-3 and -9 expression in a DSS induced model of colitis [189]. Calcium supplementation has shown benefit in reducing epithelial permeability and inflammation in the intestine through reduced expression of MMP-9, -10, and -13 in HLA-B27 transgenic rat model of colitis [190].

The pineal gland product melatonin is a known scavenger of free radicals and has anti-inflammatory effects in experimental colitis [191]. In certain instances, NO and peroxynitrite can modulate MMP-9 expression and activity [162] and it is likely that the antioxidant properties of melatonin could reduce colon gelatinase expression in DNBS induced colitis [192]. Further investigation shows that it can mediate NF-*κ*B, STAT-3, IL-17, Cox-2, nuclear erythroid 2-related factor 2, connective tissue growth factor, and MMP-9 [193] and its contribution to IBD has been summarised recently [194].

Endogenous fatty acids are known gelatinase inhibitors [195] and polyunsaturated fatty acids were recently shown to be anti-inflammatory in the gut through decreased Cox-2 and MMP-9 expression [196]. Orally administered docosahexaenoic acid had comparable efficacy to sulfasalazine in DSS induced colitis but with stronger inhibition of MMP-3, -10, and -13 [197].

## 8. MMPs as Biomarkers for IBD

The association of MMPs with IBD is now widely accepted. MMP expression or protein levels are now a standard read-out for inflammation in the experimental models described previously. There is an unmet need for additional biomarkers to assess the progression of the disease or identification of flares without the need for invasive, time consuming, or expensive imaging techniques. Here we review the recent studies regarding the potential and usefulness of MMPs as markers for inflammation in IBD.

Faecal MMP-9 levels were reported to correlate with the overall Mayo and endoscopic scores, serum CRP, and faecal calprotectin levels in UC patients [198]. As a biomarker, faecal MMP-9 also has potential in recognising severity of pouchitis and, to a lesser extent, CD where correlation with the SES (simple endoscopic score) CD was not statistically significant but overall correlations were better than calprotectin [199]. However, neither faecal calprotectin nor faecal MMP-9 can differentiate between* Clostridium difficile* induced and a natural relapse in IBD [200].

Serum MMP-9 correlates with disease activity in UC and CD and levels were found to be higher in UC. As a result, this may aid in differentiation between UC and CD where serum MMP-9 was more effective than CRP levels [127]. Neutrophil secreted MMP-9 is often complexed with NGAL and circulating levels of NGAL or NGAL/MMP-9 complex have been associated with breast cancer [21] and kidney disease [201]. This complex has recently been described as a potential biomarker for mucosal healing in UC where infliximab reduced serum levels, predictive of mucosal healing [202]. Immunohistochemical staining of colonic biopsy samples for paediatric onset UC patients showed that MMP-9 levels correlated with the histological measures of inflammation but not with any other marker of disease [203]. Urinary gelatinase levels have also been found to be independent predictors of IBD in paediatric patients [204] and MMP-3 and MMP-9 serum levels were found to be potentially useful diagnostics of disease activity in children with UC [205].

A gene expression approach was used to assess differences between responders and nonresponders to corticosteroid treatment in severe paediatric UC. MMP-8 was strongly upregulated in nonresponders compared to those patients who had responded to treatment. This effect was believed to be due to the inhibitory effect of methylprednisolone on IL-8, a known inducer of MMP-8 [206].

Microarrays were used to assess the potential of biomarkers in peripheral blood as diagnostic indicators for IBD. MMP-9 was found to be upregulated in IBD patients which corresponded to the combined epithelial and lamina expression in biopsy samples. MMP-9 was identified in this study as one of the top 5 peripheral blood transcripts which could be used in combination to diagnose UC or CD [207]. A large study investigated useful and appropriate biomarkers in IBD and their correlation with the Mayo score in UC or with the ileocolonoscopy (ICO), computed tomography enterography (CTE), or combined ICO-CTE score in CD patients as measures of inflammation. The study concluded that combined faecal calprotectin and serum MMP-9 best predicted inflammation in UC and combination of faecal calprotectin, serum MMP-9, and serum IL-22 best correlated with ICO-CTE score in CD [208].

The expression pattern of the TIMPs often mirrors that of the MMPs and so the level of inflammation. Several studies have investigated the potential of also measuring TIMP levels as readouts of inflammation severity. MMP-1 and TIMP-1 are well known to be upregulated in UC and colonic expression was found to correlate with disease severity. The fact that plasma levels of these proteins correlated well with the mucosal expression was of potential clinical interest [40]. The intestinal expression of MMPs and TIMPs in CD patients was measured before and following immunosuppressive treatment and found that the histological score correlated positively with neutrophil MMP-9, MMP-26, and macrophage TIMP-1. Calprotectin levels followed a similar trend to expression of stromal MMP-26, TIMP-1, and -3. Crohn's disease endoscopic index of severity (CDEIS) value correlated positively with macrophage TIMP-1 and stromal TIMP-3 and negatively with epithelial TIMP-3 which also negatively correlated with C-reactive protein values (CRP) [177]. The levels of MMPs and TIMPs were measured by immunohistochemistry only, and neither serum nor plasma levels were assesed. In paediatric IBD, serum MMP-7 mirrors the disease activity and, together with TIMP-1 expression, is a measure of response to glucocorticoid therapy but to a lesser extent, anti-TNF therapy [178].

## 9. Concluding Remarks

Following recognition of the limited understanding of the MMPs in earlier trials, the field has grown enormously to discover more roles for the proteases. The fact that the expression and activity of the MMPs is upregulated in colitis and IBD has been confirmed through numerous animal model experiments and analysis of IBD patient biopsies. This has led to significant research into the use of MMPs as biomarkers for the severity of inflammation in the colon where results appear promising.

More studies continue to identify the primary cellular sources of the MMPs in the colon and triggers of their transcriptional upregulation or activation. A definitive understanding of cell types and signals involved in MMP upregulating will allow us to target the pathologically upregulated enzymes.

It is clear from the range of MMP substrates and degradomic experiments that the functions of MMPs in IBD go far beyond the simple digestion of ECM proteins. In recent years, more and more studies highlight the role of MMPs in regulation of the immune response through activation or inhibition of cytokines and chemokines, for example, the activation of TNF-*α* which is exciting given the established role of this cytokine in the pathogenesis of IBD [110]. MMPs have the ability to regulate both pro- and antiangiogenic factors which may contribute to the pathogenesis of IBD or mucosal healing. The proteases have a similar role in epithelial homeostasis through control of tight junction proteins, goblet cell differentiation and degradation of ECM proteins. These studies are vital to understand the contribution of specific MMPs to a range of processes that make up IBD. However, the potential to therapeutically exploit these discoveries raises several questions. Paramount to the discussion of MMP inhibition is the potential knock on effect of the inhibition. Blocking a single enzyme will affect activation of other proteases and its considerable list of substrates which is likely to disrupt physiological processes. Delineating these activation pathways, completing substrate lists, and understanding the contexts where upregulation of the protease is pathological will remain relevant questions before MMP inhibition becomes a therapeutic option in the clinic. Therefore, while new functions emerge for the MMPs, it will be crucial to understand the setting where an MMP is promoting or inhibiting wound healing, pro- or antiangiogenic, or inhibiting or amplifying the immune response. As answers to these questions emerge, we can be more confident that targeted inhibition of the MMPs could be of benefit in treating IBD.

## Figures and Tables

**Table 1:** Recently described roles for MMPs in IBD.

MMP	Role in IBD	Reference
MMP-1	Prevention of fibrosis	[145]
MMP-2	Generation of antiangiogenic factors, maintenance of epithelial barrier function, and prevention of fibrosis	[119, 135, 145]
MMP-3	Generation of endostatin	[118]
MMP-7	*α*-defensin activation, chemokine expression, wound healing, and generation of endostatin	[105, 106, 118]
MMP-8	Neutrophil infiltration	[18]
MMP-9	Chemokine expression, neutrophil infiltration, generation of anti-angiogenic factors, VEGF-A processing, decreased goblet cell differentiation, and prevention of fibrosis	[18, 107, 118, 119, 125, 134, 145]
MMP-10	Wound healing,	[19]
MMP-13	Activation of TNF-*α* and generation of endostatin	[111, 118]
MMP-20	Generation of endostatin	[118]

**Table 2:** Recently reported MMP inhibitors in models of intestinal inflammation. Novel MMP inhibitors, plant extracts tested in IBD models, and existing IBD therapies are included where MMP-9 expression or activity has been measured.

MMP inhibitor	MMPs inhibited	Model	Reference
RO28-2653	MMP-2, -9	DSS (mouse) (acute)	[163]
Ilomastat	MMP-1	TNBS (rat)	[173]
Minocycline	MMP-2, -3, -9, -13	DSS or TNBS (mouse)	[172]
Etiasa (mesalazine)	MMP-2	TNBS (rat)	[30]
Irsogladine maleate	MMP-2	DSS (mouse)	[209]
Infliximab	MMP-1, -2, -3, -9, -13	Human CD (serum and biopsy)	[174, 175]
CC-10004	MMP-3	Mononuclear cells from human CD	[165]
Nitrate-barbiturates	MMP-9	Cytokine stimulated Caco-2 cells	[161]
Auraptene	MMP-7, -2, -9	DSS (mouse)	[180]
coumarin 4-methylesculetin	MMP-9	TNBS (rat)	[181]
Tris(methoxymethoxy)chalcone	MMP-7	TNF-*α* stimulated HT-29 cells	[182]
Curcumin	MMP-3	Cultured colonic myofibroblasts from IBD patients	[183]
Phenylpropanoid glycosides: teupolioside and verbascoside	MMP-2, -9	DNBS (rat)	[184, 185]
Neovastat	MMP-9	TNBS (rat)	[188]
*Α*lpha-lipoic	MMP-9	DSS (mouse)	[210]
Cordyceps militaris	MMP-3 and -9	DSS (mouse)	[189]
Calcium (CaHPO_4_)	MMP-9, -10, -13	HLA-B27 transgenic rat	[190]
